# Dehydration Stress Memory Genes in Tomato (*Solanum lycopersicum* L.)

**DOI:** 10.3390/ijms27146187

**Published:** 2026-07-10

**Authors:** Monther T. Sadder, Abdullah A. Alsadon, Bayan S. Alkharabsheh, Anas Musallam, Abdulsalam M. Alnajjar, Lana W. Al-Qadumii

**Affiliations:** 1 Department of Horticulture and Crop Science, School of Agriculture, University of Jordan, Amman 11942, Jordan; radwan_bayan22@yahoo.com (B.S.A.); abd.alsalam93@gmail.com (A.M.A.); 2 Department of Plant Production, College of Food and Agricultural Sciences, King Saud University, P.O. Box 2460, Riyadh 11451, Saudi Arabia; alsadon@ksu.edu.sa; 3 Biotechnology Research Directorate, National Agricultural Research Center, Baq’a 19381, Jordan; anas_musllam@yahoo.com; 4 Department of Agricultural Biotechnology and Genetic Engineering, Faculty of Agricultural Technology, Al-Ahliyya Amman University, Amman 19328, Jordan; lana_qadumii@hotmail.com

**Keywords:** abiotic, drought, physiology, transcriptional memory, tomato

## Abstract

Drought is among the most serious abiotic stresses affecting tomato production worldwide, especially under climate change. Plants exposed to repeated drought events may develop stress memory, allowing them to respond more efficiently to subsequent stress exposure. In this study, physiological and transcriptomic tools were combined to investigate dehydration stress memory in tomato (*Solanum lycopersicum* L.). Tomato plants were subjected to two consecutive drought stresses separated by a recovery stage, where control (C), first stress (S1), rehydration (H), and second stress (S2) stages were analyzed. Physiological measurements showed progressive reductions in relative water content (RWC) and PSII activity under drought stress, while proline accumulation was significantly increased during the second stress stage, indicating memory-associated adaptive responses. RNA sequencing revealed dramatic transcriptome reprogramming with thousands of differentially expressed genes (DEGs) across stress stages. Hierarchical clustering identified 30 distinct expression patterns among revealed DEGs, including clusters associated with transcriptional memory, adaptive responses, metabolic adjustment, and recovery processes. Several memory-associated clusters were enriched with transcription factors, signaling proteins, osmolyte-related genes, and reactive oxygen species detoxification enzymes. Gene Ontology analysis highlighted significant enrichment of pathways related to photosynthesis, response to water deprivation, ABA signaling, oxidative stress, carbohydrate metabolism, and chromatin organization. Recovery-associated expression of histone and chromatin remodeling genes indicates a potential involvement of epigenetic-related regulatory processes in dehydration stress memory in tomato. Tomato plants respond to repeated dehydration stress through coordinated physiological, metabolic, transcriptional, and epigenetic adjustments that improve stress adaptation. The identified candidate memory genes may provide useful targets for future breeding programs aimed at enhancing drought tolerance in tomato.

## 1. Introduction

Tomato is one of the most economically important vegetable crops and is widely cultivated globally as a high-value cash crop. Tomato cultivation is increasingly expanding, with an annual increase of around 1.2% worldwide [1]. However, climate change-associated abiotic stresses, particularly drought, have become major constraints limiting tomato productivity, leading to substantial yield losses and increased vulnerability to pests and diseases [2]. To address these challenges, extensive tomato genetic resources have been conserved in gene banks, research institutions, and seed companies, collectively comprising tens of thousands of accessions [3]. Comprehensive physiological and phenological characterization of this germplasm is essential for the development of climate-resilient tomato ideotypes suitable for future environmental conditions [4,5]. Consequently, recent research efforts have increasingly focused on the functional valorization of these genetic resources to develop improved breeding lines with enhanced stress resilience [6].

At the molecular level, plant adaptation to environmental stress is largely mediated by transcriptional reprogramming. Successful acclimation involves coordinated regulation of stress-responsive genes, whereas failure to adjust transcriptionally often results in stress sensitivity [7]. Accordingly, gene expression profiling has become a powerful approach to distinguish tolerant and sensitive plant responses under adverse conditions [8]. In particular, transcriptional regulation is largely governed by stress-responsive genes, including a wide range of DNA-binding transcription factors that orchestrate downstream adaptive pathways [4,9].

Beyond single stress events, plants can exhibit a phenomenon known as stress priming, where prior exposure to a stress modifies physiological, biochemical, and transcriptional responses to subsequent stress exposure of similar type. This enhanced responsiveness is often described as a form of “stress memory,” which may improve plant performance under recurrent environmental challenges [10,11]. Genome-wide transcriptomic analyses in Arabidopsis have demonstrated that repeated dehydration stress induces distinct transcriptional memory patterns, revealing complex regulatory states underlying stress recall mechanisms [12,13]. These findings highlight that stress memory is not a uniform response but instead involves multiple transcriptional response categories that depend on stress history.

Despite these advances, the existence and mechanisms of drought stress memory in tomato remain poorly understood. Only a limited number of studies have addressed this concept in tomato. For instance, differential expression of the DREB2 subfamily has been associated with salinity-related memory responses [14], while seed priming with salt has been shown to influence tomato agronomic performance [15]. In addition, several studies have investigated heat stress memory and its physiological consequences in tomato [16,17]. Regarding drought-related memory responses, only a few reports are available, including studies on combined drought and ultrasound treatments [18], physiological assessments of repeated drought exposure [19], and the use of GABA priming to induce drought-associated memory effects in tomato [20]. However, a comprehensive transcriptome-wide analysis of drought stress memory under a defined stress–recovery–stress framework in tomato is still lacking.

Therefore, this study was designed to investigate drought stress memory-associated transcriptional and physiological responses in tomato using a controlled repeated dehydration system (stress–recovery–stress). Specifically, we aimed to (i) characterize physiological responses during sequential dehydration cycles, (ii) identify differentially expressed genes associated with repeated stress exposure, and (iii) determine whether prior dehydration modifies the transcriptional response to subsequent stress. This study provides a systematic framework for understanding drought stress memory-associated regulation in tomato.

## 2. Results

### 2.1. Physiological Parameters

**Figure 1:**
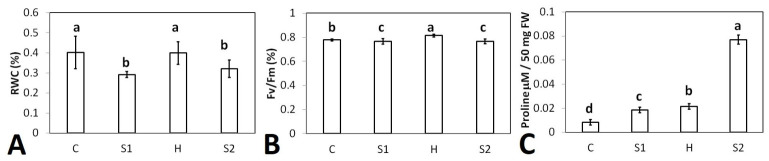
Comparison of RWC (**A**), PSII activity (**B**) and proline content (**C**) in tomatoes in control (C), first stress (S1), rehydration (H) and second stress (S2) stages. Data represent means ± SD. Different letters indicate significant differences between treatments as determined by LSD (*p* < 0.05), n = 5.

Two successive dehydration stresses separated by a short rehydration period (H stage) were applied to assess physiological and molecular stress memory responses in the studied of tomato cultivar. Relative water content (RWC) significantly decreased during the first stress (S1) to approximately ~30%, accompanied by visible leaf wilting (Figure 1A). After the 4-day rehydration period (H), plants recovered turgor, with leaf expansion restored and RWC increasing to ~40%, comparable to control levels, indicating physiological recovery prior to the second stress exposure.

During the second dehydration stress (S2), RWC again declined to levels similar to S1, confirming reproducible stress induction. Photosystem II (PSII) efficiency showed only a moderate reduction under both S1 and S2, indicating partial maintenance of photochemical performance under dehydration (Figure 1B).

Proline content increased slightly but significantly during S1 and remained relatively stable during the rehydration phase. However, a markedly stronger accumulation was observed during S2 compared to S1, suggesting a memory-associated metabolic response consistent with a primed or memory-like effect following prior stress exposure (Figure 1C).

Although dehydration stress was re-imposed during S2, RWC values during S2 were comparable to those observed during S1, indicating a similar level of water deficit between both stress cycles. Therefore, the differential responses observed during S2 are unlikely to be explained solely by greater stress severity.

### 2.2. RNA Sequencing and Transcriptome Data Analysis

**Figure 2:**
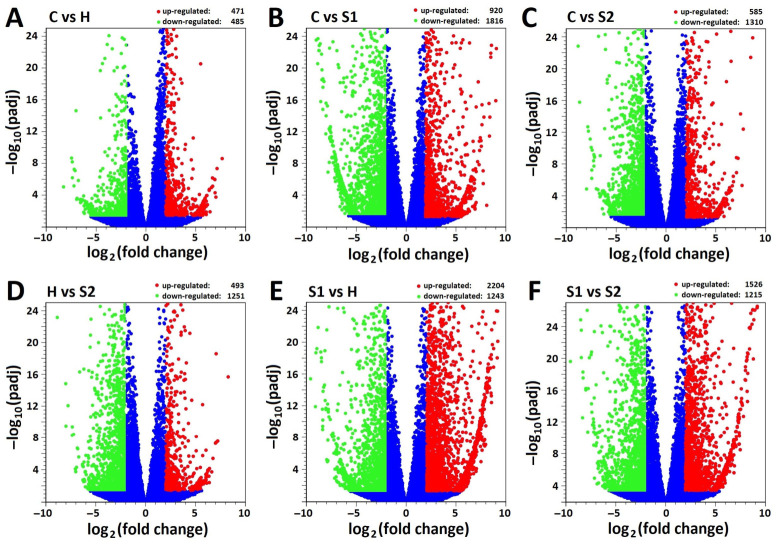
Global analysis of differentially expressed genes (DEGs) with fold change ≥ 2. (**A**) Volcano plot of DEGs for C (control) vs. H (rehydration). (**B**) Volcano plot of DEGs for C (control) vs. S1 (first stress). (**C**) Volcano plot of DEGs for C (control) vs. S2 (second stress). (**D**) Volcano plot of DEGs for H (rehydration) vs. S2 (second stress). (**E**) Volcano plot of DEGs for S1 (first stress) vs. H (rehydration). (**F**) Volcano plot of DEGs for S1 (first stress) vs. S2 (second stress). The abscissa shows the fold change difference in the expression of genes in different comparison groups, and the vertical coordinates indicate the adjusted *p*-values for the differences in expression. Genes without significant differences (adjusted *p*-value ≤ 0.05) are indicated by blue dots below the threshold value (1.3). The up-regulated genes are represented by red dots, and the down-regulated genes are represented by green dots.

The aim of this part of the study was to investigate transcriptome changes in tomato under induced drought stress stages that facilitate memory genes to operate as they would be expected in multiple stress conditions. The cDNA libraries were synthesized for tomato seedlings under the control (C), first stress (S1), rehydration (H) and second stress (S2) stages. The generated total reads (all replicates) were around 95, 87, 94 and 92 million reads for C, S1, H and S2, respectively. The PCA of all four stages revealed good proximity for all biological replicates in each sample (Supplementary Figure S1). The analysis of differentially expressed genes (DEGs) is an invaluable tool to identify genes that may be responsible for drought tolerance in the tomato. DEGs were identified from the transcriptome data, with a *p*-value ≤ 0.05 and with a threshold of Log2FC ≥ 2 (Figure 2). In the case of the C vs. H comparison, a total of 956 DEGs were identified, 471 with up-regulated expression and 485 with down-regulated expression (Figure 2A). However, when comparing C vs. S1 and S2, 920 and 585 DEGs were up-regulated in C, while 1816 and 1310 DEGs were up-regulated in S1 and S2, respectively, showing the huge response to drought stress as compared to the control plants (Figure 2B,C). A total of 1251 and 2204 DEGs were up-regulated in S1 and S2 as compared to the rehydration stage, respectively (Figure 2D,E). DEGs related to memory are clear when comparing S1 vs. S2, where the latter showed up-regulation of 1215 DEGs (Figure 2F). qPCR data confirm the RNAseq data, as they showed similar trends in gene expression (Supplementary Figure S2).

**Figure 3:**
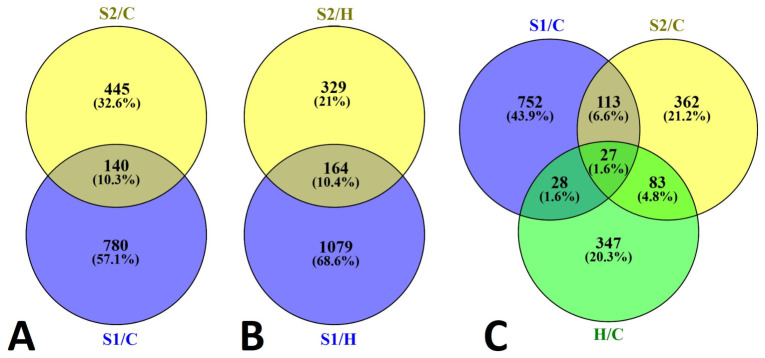
Venn diagrams of DEGs with 2-fold expression and above for tomato ‘Revenant’ under S1 and S2 compared to the control (**A**), under S1 and S2 compared to rehydration stage (**B**) and under S1, S2 and H compared to the control (**C**).

Using Venn diagrams (Figure 3), multiple comparisons were made for overlapping DEGs and unique ones. In the first comparison between the DEGs during S1 and S2 drought stresses as compared to the control (Figure 3A), it was evident that 140 genes are shared between the two stresses; nonetheless, each stress showed up-regulation of unique genes (780 for S1 and 445 for S2). On the other hand, and as compared to the rehydration stage (H), 164 DEGs were in common while 1079 and 329 DEGs were differentially expressed in S1 and S2, respectively (Figure 3B). The final comparison, similar to the first one, was compiled in addition to H compared to the control, where still the majority of DEGs were unique to each stage (Figure 3C).

Several thousand DEGs exhibited significant differential expression across drought stress and recovery (adjusted *p*-value < 0.01, log2(fold change) ≥ 2). On the basis of similar kinetic patterns of expression, all DEGs were classified into a total of thirty unique gene expression patterns (clusters) (Figure 3). Differentially expressed genes (DEGs) in control (C), first stress (S1), rehydration (H) and second stress (S2) stages were used to reveal these 30 clusters. Each cluster was built from a large number of DEGs, ranging from 69 up to 1562 genes.

**Figure 4:**
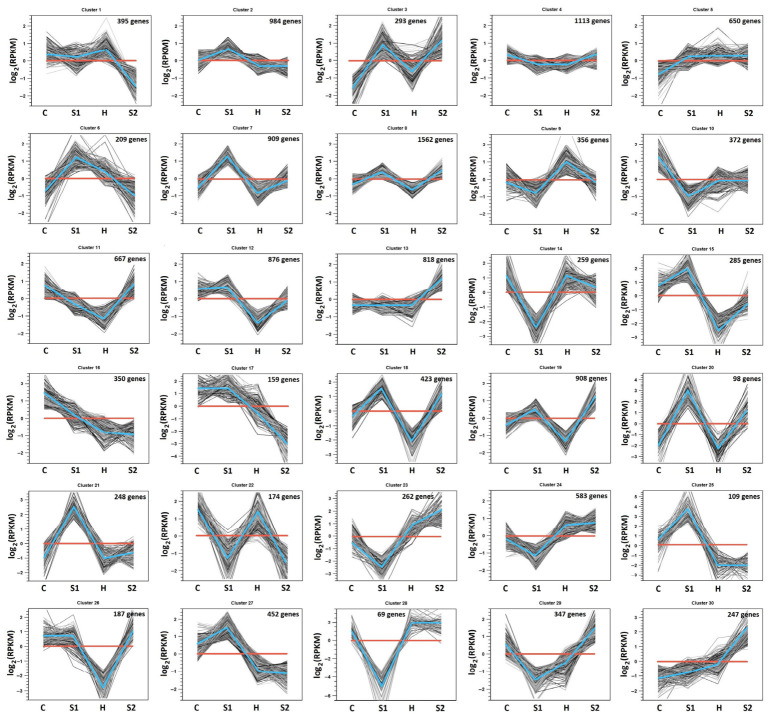
Gene expression patterns obtained by hierarchical clustering. Differentially expressed genes (DEGs) in tomato were categorized into 30 clusters (cluster number is located above each panel). Gray lines show the relative expression levels of DEGs in the cluster in control (C), first stress (S1), rehydration (H) and second stress (S2) stages. Blue lines show the average values for each relative expression cluster. Red lines represent the baseline. Levels of gene expression were represented along the y-axis as log2(RPKM), and stages were represented along the x-axis.

A total of thirty gene expression patterns were resolved, each with different gene numbers (Figure 4). Recognized patterns along the four stages (C, S1, H and S2) and the proposed function alongside major genes in the cluster and their role are listed in Table 1 and summarized in Table 2.

Several clusters showed enhanced or repeated induction during the second stress stage, which is consistent with gene memory. Four major types could be identified (Table 1); Type I memory refers to genes that show an enhanced reactivation upon repeated stress (S2 compared to S1), reflecting a priming effect where prior drought exposure strengthens transcriptional responses, particularly in stress-related transcription factors, ABA/ROS signaling components, and detoxification enzymes. Type II memory describes genes that exhibit an attenuated response during subsequent stress exposure, indicating a dampened transcriptional activity that likely represents an energy-saving strategy through reduced signaling and stress-response investment. Metabolic memory encompasses genes with progressively increased or sustained expression across stress cycles, mainly associated with carbohydrate, lipid, and energy metabolism, supporting continuous osmotic adjustment and metabolic reprogramming under repeated drought conditions. Recovery or epigenetic memory involves genes related to chromatin organization, histone regulation, and DNA repair, which are modulated during recovery phases and are thought to contribute to stress-induced epigenetic resetting or maintenance of transcriptional states that influence future stress responsiveness. These clusters captured both transient and persistent transcriptional responses, indicating that the studied tomato cultivar under the tested experimental conditions exhibits complex regulatory behavior beyond a simple stress–recovery cycle. Multiple transcription factor family members were represented across these clusters, including 49 WRKY-related genes (e.g., Solyc03g104810, Solyc07g051840 in cluster 3) and 26 ethylene-responsive transcription factor (ERF)-related genes (e.g., Solyc01g065980, Solyc03g123500 and Solyc06g063070 in cluster 7), together with signaling components such as receptor-like kinases and calcium-dependent protein kinases, suggesting broad transcriptional and signaling regulation during repeated dehydration stress. Other clusters showed attenuated responses, suggesting adaptive regulation to reduce metabolic cost. Metabolic memory genes include osmolyte biosynthesis and carbohydrate metabolism. Moreover, certain clusters were enriched in histones and chromatin-associated proteins with up-regulation during the rehydration stage, which indicates epigenetic control over dehydration stress memory genes. Each experimental stage was characterized by a unique set of differentially expressed genes. The S1 stage (memory initiation) showed activation of signaling genes (CDPKs and calmodulin), while the H stage (memory storage) showed up-regulation of chromatin remodeling, metabolic adjustment and signal retention-related genes. On the other hand, after the second drought stress stage, faster TF activation coupled with optimized metabolism and reduced energy cost was evident.

The clusters (3, 7, 19, 21 and 30) showed up-regulation in the second stress (S2) compared to the first stress (S1). They presented expression profiles which are characteristic for transcriptional memory, where a stress event primes genes for faster activation in a subsequent stress S2 (Type I memory genes). On the other hand, clusters (6, 10, 15, and 16) showed attenuated expression during S2, despite strong induction during S1 (Type II memory genes). An additional unique profile was evident in clusters (4, 5, 8, 13, 20, 23 and 29), where progressive expression was detected across stress cycles, indicating metabolic memory (Table 1). They involve genes for carbohydrate metabolism (invertases), organic acid metabolism (malic enzyme), and osmolyte biosynthesis (proline and betaine pathways). Moreover, certain clusters (9, 11, 14, 22, 27, and 28) were enriched in photosynthesis-related and ribosomal genes, which were down-regulated during stress conditions. Notably, clusters (11, 18, and 22) peaked during rehydration stage, indicating the importance of the recovery stage in gene regulation. Furthermore, enrichment of chromatin regulators may help establish epigenetic modifications during recovery, providing persistent regulation of transcriptional memory. Along multiple clusters, genes involved in signal transduction, including receptor-like kinases (RLKs), calcium-dependent protein kinases (CDPKs), and calmodulin-related proteins, were evident and facilitate signal perception during repeated stress. Additionally, major clusters were enriched in reactive oxygen species (ROS) detoxification genes, such as ascorbate peroxidase, glutathione S-transferases, and peroxidases, indicating that redox homeostasis is a major component of drought memory.

**Figure 5:**
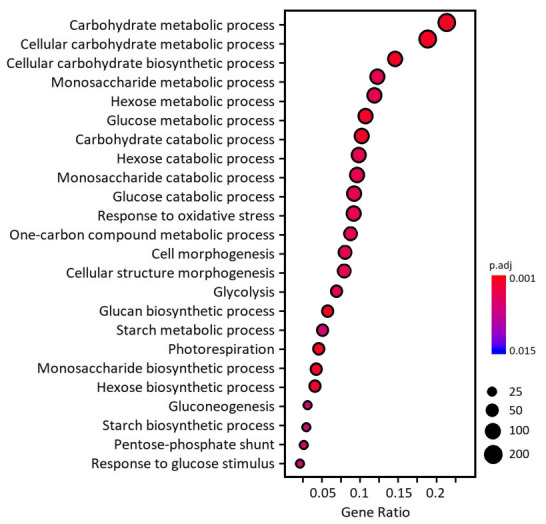
Bubble plot of GO biological process enrichment analysis results for DEGs in cluster 4. The *x*-axis indicates the proportion of genes per functional term. The *y*-axis indicates the annotated terms of gene enrichment. The circle size represents the number of genes: the larger the circle, the higher the number of genes. The circle color represents the adjusted *p*-value: the redder the color, the higher the degree of gene enrichment.

Gene Ontology (GO) enrichment analysis of differentially expressed genes showing distinct expression patterns across repeated dehydration stress and recovery stages revealed significant enrichment of biological processes related to stress adaptation, cellular regulation, and metabolic adjustment. Among the most enriched categories in cluster 4 were carbohydrate metabolism and biosynthesis, with values of ~0.22 and ~0.19, respectively (Figure 5), with strong statistical significance (low adjusted *p*-values). These findings suggest that dehydration stress memory genes are closely associated with maintaining energy use efficiency and improving drought tolerance during recurrent stress exposure.

**Figure 6:**
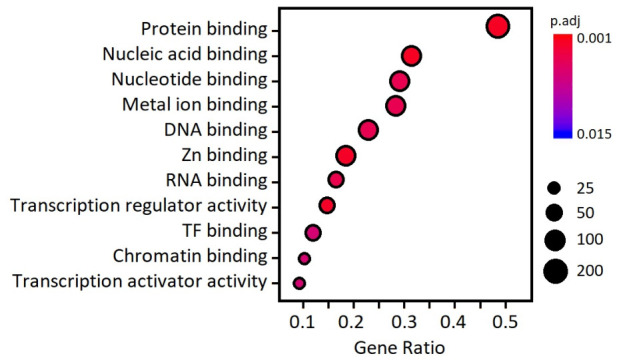
Bubble plot of GO biological process enrichment analysis results for DEGs in cluster 11. The *x*-axis indicates the proportion of genes per functional term. The *y*-axis indicates the annotated terms of gene enrichment. The circle size represents the number of genes: the larger the circle, the higher the number of genes. The circle color represents the adjusted *p*-value: the redder the color, the higher the degree of gene enrichment.

On the other hand, GO biological process enrichment analysis in cluster 11 revealed major epigenetic genes related to DNA and histone-binding proteins (Figure 6).

Several signaling and regulatory pathways were also significantly enriched, including ABA signaling and signal transduction, indicating an important role for hormonal and signaling networks in stress memory establishment and maintenance. In addition, enrichment of oxidative stress and DNA repair categories suggests activation of protective mechanisms that minimize cellular damage caused by abiotic stress conditions.

Metabolic processes such as carbohydrate metabolism and lipid metabolism were also overrepresented, reflecting metabolic reprogramming and energy redistribution during stress adaptation. Furthermore, enrichment of translation, protein catabolism, and chromatin organization indicates that transcriptional and post-transcriptional regulation, protein turnover, and epigenetic modifications contribute substantially to stress memory responses in tomato. Overall, these results demonstrate that tomato dehydration stress memory genes participate in coordinated physiological, metabolic, and regulatory pathways that enhance plant adaptation to repeated abiotic stress conditions.

## 3. Discussion

A memory gene describes an expression pattern, where the level in a first-applied stress affects the level in a subsequent stress interrupted by a recovery period [7]. The present study showed that drought memory in tomato is controlled by an integrated regulatory network, including signaling, transcriptional activation, metabolic pathways, and epigenetic remodeling. Several distinct gene clusters were identified, where the investigated tomato cultivar modifies its response based on prior stress exposure, supporting transcriptional priming, a major mark of drought memory. Similar regulatory frameworks have recently been described as central components of plant stress memory, where repeated stress exposure leads to long-lasting transcriptional reprogramming mediated by chromatin and signaling networks [21,22,23,24]. Our results extend these findings to tomato, confirming that transcriptional memory is a conserved feature across diverse plant species.

The present study focused on selected physiological indicators including relative water content, PSII efficiency, and proline accumulation to characterize plant responses during repeated dehydration stress. Although these parameters provided evidence of dehydration and recovery dynamics, additional measurements such as stomatal conductance, transpiration rate, leaf water potential, ABA accumulation, oxidative stress markers, and antioxidant enzyme activities would provide a more comprehensive characterization of physiological adaptation during repeated stress exposure. Future studies integrating these parameters will help further distinguish stress memory mechanisms from cumulative stress effects.

A key feature of memory-positive clusters is the enrichment of transcription factors such as WRKY, ERF, and bZIP, along with signaling components including RLKs and CDPKs. WRKY transcription factors are known to regulate drought-responsive genes by binding to W-box cis-elements (TTGACC/T) in the promoters of target genes, thereby directly modulating transcriptional activation during stress re-exposure and contributing to transcriptional priming [25]. In several cases, WRKY proteins act in coordination with ABA signaling through functional interplay with ABA-responsive element-binding factors (AREB/ABF), integrating ABA-dependent and ABA-independent pathways to fine-tune stress-inducible gene expression [26]. Similarly, ERF transcription factors regulate drought and dehydration responses through binding to DRE/CRT elements and are key components of ethylene–ABA crosstalk, which stabilizes stress-responsive transcriptional programs during repeated stress exposure [27]. The strong enrichment of bZIP family members further supports ABA-dependent regulation, as AREB/ABF proteins are phosphorylated by SnRK2 kinases and activate ABA-responsive promoters [26]. Calcium signaling components, particularly CDPKs and calmodulin-related proteins, likely act upstream as early stress sensors that transduce Ca^2+^ signatures into phosphorylation cascades, ultimately activating these transcriptional networks, consistent with the decoding of calcium signatures in plant abiotic stress signaling [28]. This aligns with previous studies showing that calcium-mediated signaling contributes to the activation of stress-responsive genes during repeated stress [29]. On the other hand, our data revealed enrichment of chromatin-related genes in recovery clusters, which may suggest a possible contribution of chromatin-related regulatory processes to dehydration stress memory. However, direct involvement of epigenetic memory mechanisms requires further experimental validation through dedicated epigenetic analyses. Histone proteins and methyl-binding domain proteins imply chromatin remodeling to maintain accessibility to drought-responsive genes in coming stress stages [30]. Recent studies increasingly demonstrate that chromatin accessibility, histone modifications, and RNA polymerase II retention represent major regulatory layers controlling stress memory establishment under repeated drought conditions [12,31,32]. The persistence of such marks in the recovery would explain “memory storage”, which can lead to faster gene activation in subsequent stress.

**Figure 7:**
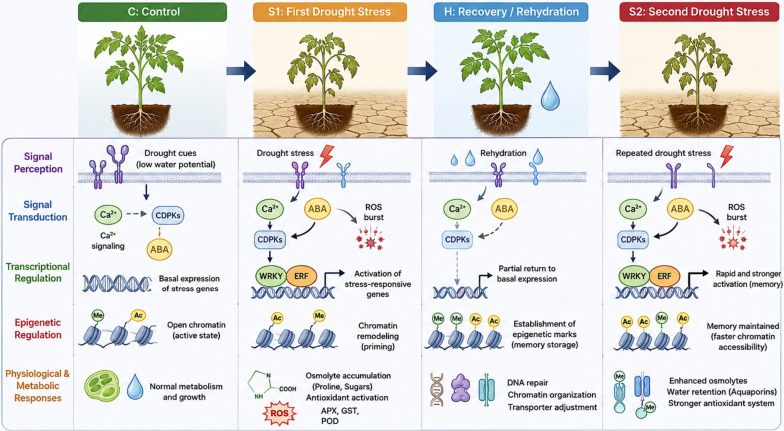
Proposed conceptual model of transcriptional dehydration stress memory in tomato based on the results of the present study. The conceptual design was developed by the authors, and the graphical illustration was generated with the assistance of ChatGPT-5.5 under the authors’ guidance and subsequently reviewed and edited by the authors. Solid arrows indicate experimentally supported relationships, whereas dashed arrows represent proposed interactions inferred from the present results.

The physiological responses observed in the tested cultivar were strongly associated with the transcriptional reprogramming detected during repeated dehydration stress (Figure 7). The progressive decline in relative water content (RWC) during S1 and particularly during S2 reflects increasing cellular dehydration, which corresponded with the enhanced expression of aquaporins, ABA-responsive genes, and signaling-related transcription factors identified in several memory-associated clusters. Aquaporins are known to regulate transmembrane water movement and contribute to cellular water balance under drought conditions, while ABA signaling coordinates stomatal closure and osmotic adjustment to minimize water loss. In parallel, the significant accumulation of proline during S2 is consistent with the enrichment of genes involved in osmolyte biosynthesis and carbohydrate metabolism, indicating that the studied genotype establishes a metabolic memory that enhances osmotic protection during recurrent stress exposure. Similar associations between proline accumulation and transcriptional activation of stress-responsive metabolic pathways have been reported in drought-stressed Arabidopsis and maize memory studies [21,22,33]. The observed reduction in PSII activity during dehydration stress was also supported by transcriptomic evidence. Although PSII activity showed only a limited physiological decline during dehydration stress, transcriptomic analysis revealed substantial down-regulation of photosynthesis-related genes, suggesting early molecular adjustment of photosynthetic pathways that may contribute to resource reallocation during repeated stress exposure. Several clusters enriched with photosynthesis-related and ribosomal genes showed marked down-regulation during stress treatments, particularly during S2. Such coordinated suppression of photosynthetic machinery is considered an adaptive strategy to reduce energy consumption and limit oxidative damage during water-deficit conditions. At the same time, enrichment of oxidative stress and ROS detoxification genes, including glutathione S-transferases, peroxidases, and ascorbate peroxidases, suggests activation of protective antioxidant systems to preserve cellular integrity. Together, these findings demonstrate that physiological drought responses in tomato are closely linked with transcriptional memory mechanisms that optimize water conservation, osmotic balance, photosynthetic adjustment, and oxidative stress protection during repeated dehydration events.

The enrichment of ABA signaling-related genes together with ethylene-responsive transcription factors suggests the possible involvement of multiple hormone-associated signaling pathways during dehydration stress memory. However, direct hormonal interactions remain to be experimentally validated through hormone quantification and functional studies. Several clusters contained ERFs, indicating that ethylene signaling may cooperate with ABA-mediated pathways during repeated stress exposure. Ethylene has been shown to regulate stress-responsive gene expression, ROS homeostasis, and stomatal behavior, particularly during prolonged or recurrent abiotic stress conditions. The simultaneous enrichment of ABA signaling genes and ERF-associated clusters suggests coordinated interaction between these hormonal pathways to fine-tune stress adaptation and memory establishment. In addition to ABA and ethylene, reactive oxygen species (ROS)-related signaling pathways were strongly represented through enrichment of detoxification enzymes and oxidative stress-responsive genes as reported in tomatoes [34]. On the other hand, auxin signaling represents an additional layer of drought-responsive transcriptional regulation alongside ABA and ERF pathways. During drought stress, elevated ABA levels can reduce free IAA while increasing IAA conjugation, thereby modulating growth–stress balance. This ABA–auxin interaction provides a mechanistic basis for coordinated hormonal control under stress [35]. Consistently, our data identified auxin biosynthesis-related DEGs in key memory clusters, suggesting a role for auxin in transcriptional memory alongside ABA and ERF signaling. ROS are increasingly recognized not only as damaging molecules but also as secondary messengers that interact with hormonal networks during stress signaling. Recent drought memory models emphasize strong integration between ABA, calcium signaling, ROS signaling, and transcription factor networks in coordinating rapid secondary stress responses [36]. Furthermore, the presence of genes associated with jasmonic acid- and salicylic acid-related transcriptional regulation suggests that multiple hormonal pathways may collectively participate in coordinating defense, metabolic adjustment, and recovery processes during drought memory. Such hormonal integration enables plants to generate highly flexible and efficient adaptive responses under fluctuating environmental conditions.

Beyond transcriptional regulation, our results highlight the importance of metabolic and physiological adaptations in drought memory. Clusters enriched in genes involved in osmolyte biosynthesis, carbohydrate metabolism, and lipid metabolism indicate that plants undergo biochemical reprogramming. Such metabolic memory can help in osmotic balance and saving energy during repeated stress, as suggested in maize and other crops [33]. The identification of aquaporins further highlights the role of water transport regulation in enhancing drought tolerance. Furthermore, repeated suppression of growth-related genes, including photosynthetic and ribosomal proteins, supports the involvement of memory in down-regulation of growth processes. Such negative regulation has been reported in earlier studies, where drought memory leads to modified stomatal responses and reduced growth under repeated stress conditions [37]. This dual regulation ensures that plants balance survival and growth under fluctuating environments.

A novel insight from this study is the identification of the recovery phase as an active stage of memory consolidation. Rather than being a passive return to baseline, recovery is characterized by the activation of chromatin remodeling, repair processes, and transport mechanisms. This finding is consistent with emerging evidence that stress memory is established during the post-stress period, when epigenetic marks are stabilized and metabolic adjustments are fine-tuned [38,39]. It also aligns with recent work in tomato showing that priming treatments can induce long-term transcriptional memory [18,20]. The recovery phase after dehydration stress represents an active regulatory window in which stress memory is consolidated rather than a passive return to homeostasis. In our system, this phase is characterized by the differential regulation of chromatin remodeling and DNA repair-related genes, indicating coordinated epigenetic reprogramming. Chromatin modifiers can either preserve stress-induced transcriptional states or reset gene expression by altering chromatin accessibility. Dynamic changes in histone marks such as H3K4me3 (activation/priming) and H3K27me3 (repression) likely contribute to transcriptional memory maintenance or erasure. In parallel, DNA repair genes suggest activation of genome maintenance pathways to resolve stress-induced oxidative damage and restore genome stability. DNA repair and chromatin remodeling are functionally linked, as repair processes recruit histone modifiers to damaged loci, integrating genome stability with transcriptional control. Together, these mechanisms define recovery as a key epigenetic decision point between memory retention and resetting of stress-responsive genes. This aligns with current models of chromatin-based stress memory, where recovery phases act as reprogramming windows shaping future stress responsiveness [12,40]. The identification of key dehydration stress memory genes in tomato has significant implications for crop breeding and stress resilience. Genes such as WRKY, ERF, aquaporins, and ROS detox enzymes represent promising targets for marker-assisted selection and CRISPR-based editing. Recent evidence suggests that exploiting stress memory-associated genes may represent an important strategy for breeding climate-resilient crops under increasingly variable drought conditions [7,41,42]. Our findings provide a similar framework for tomato, highlighting candidate genes and pathways that could be leveraged for enhancing resilience.

The enrichment of chromatin-associated proteins and histone-related genes during the rehydration stage strongly suggests that epigenetic regulation contributes to dehydration stress memory in tomato. Recovery-associated clusters contained genes involved in chromatin organization, nucleosome assembly, and transcriptional regulation, indicating that the recovery phase functions as an active period of memory consolidation rather than merely a return to pre-stress conditions [41,43,44]. Previous studies in Arabidopsis demonstrated that dehydration stress memory genes are associated with persistent histone modifications, particularly H3K4me3, which maintains transcriptionally permissive chromatin states following initial stress exposure [13,21]. Similar mechanisms may operate in tomato, where chromatin remodeling during recovery could facilitate faster and stronger activation of stress-responsive genes during subsequent dehydration events.

In addition to histone modifications, other epigenetic processes such as DNA methylation, chromatin accessibility, and RNA polymerase II retention may contribute to maintaining transcriptional memory. The repeated activation of specific transcription factor families, including WRKY, ERF, and bZIP proteins, may depend on sustained epigenetic marks that preserve the accessibility of promoter regions under recurrent stress. Moreover, the enrichment of recovery-stage genes related to DNA repair and chromatin maintenance suggests that epigenetic stability is critical for preserving genome integrity during repeated dehydration cycles. Collectively, these findings support the hypothesis that tomato drought memory involves coordinated epigenetic reprogramming that enables plants to retain information from previous stress encounters and respond more efficiently to future dehydration stress.

Although this study provides important insights into dehydration stress memory in tomato, it was conducted using a single cultivar, and drought responses may vary among genotypes [7]. Therefore, the identified transcriptional patterns and candidate genes should be considered specific to the studied genotype under the tested conditions. Future studies involving drought-tolerant and drought-sensitive tomato genotypes are needed to validate the broader applicability of these findings.

## 4. Materials and Methods

### 4.1. Plant Materials

Tomato (*Solanum lycopersicum* L.) cultivar ‘Revenant’ (Bayer Holland B.V., Bergschenhoek, The Netherlands) was used in this study. Seeds were germinated in peat moss tray cells (50 mL each) and were irrigated every other day for three weeks. Thereafter, seedlings were transferred to new pots (2 L) with mixed medium (1 sand:1 peatmoss:1 soil) and kept for three more weeks. The photoperiod was 12 h light and 12 h dark.

### 4.2. Dehydration Stress

Experimental plants were arranged in a completely randomized design (CRD) with five biological replicates. All plants were grown under controlled greenhouse conditions (25 ± 2 °C). The experiment followed a sequential stress exposure design in which the same set of plants was subjected to four stages: control (C), first drought stress (S1; 5 days of water withholding), recovery/rehydration (H; 4 days of rewatering), and second drought stress (S2; additional 5 days of water withholding applied to the same plants). Thus, S1, H, and S2 represent successive physiological states of the same experimental units, enabling assessment of drought stress memory responses.

### 4.3. Physiological Measurements

Photosynthesis (PSII) activity was determined for leaves of C, S1, H and S2 seedlings by measuring transient chlorophyll fluorescence using Handy PEA (Hansatech, Pentney, UK) with an excitation light energy of 3000 μmol m^−1^ s^−1^. Leaf relative water content (RWC) was measured as described [45] with minor modifications, where young leaf disks (around 5 cm × 5 cm) were immersed in deionized water in PP vessels secured with cover lids and incubated for 24 h at RT in the dark. Moreover, proline content was determined as described earlier [46]. Data were analyzed using one-way analysis of variance (ANOVA), and treatment means were compared using the Least Significant Difference (LSD) test at *p* ≤ 0.05. Results are presented as mean ± standard deviation (SD).

### 4.4. RNA Isolation and cDNA Preparation

Plant leaf tissues were collected from all experimental stages; C: control, S1: first stress, H: rehydration and S2: second stress. Three biological replicates were taken for each stage. Total RNA from tomato leaves was isolated using the GF-1 Total RNA Extraction Kit (Vivantis, Selangor, Malaysia) according to the manufacturer’s protocol. All materials were treated with RNase Away (RNase Away, Molecular Bio Products, San Diego, CA, USA) to avoid the degradation of RNA by RNase. The RNA (5 µg) was used for the subsequent preparation of cDNA for each sample using the SMARTer cDNA Synthesis kit (Clontech, San Jose, CA, USA). The reaction was performed in 0.2 mL nuclease-free PCR tubes (Axgen, Stanford, CA, USA) according to the manufacturer’s instructions. The tubes were placed in a thermal cycler (Veriti 96-well; Applied Biosystems, Singapore) according to the manufacturer’s protocol.

### 4.5. RNA Sequencing and Transcriptome Data Analysis

RNA sequencing was performed at Macrogen (Seoul, Republic of Korea). RNA libraries were prepared from three biological replicates per treatment (C, S1, H, and S2) using Illumina-compatible barcoded adapters (Illumina, San Diego, CA, USA). Paired-end sequencing (2 × 101 bp) was performed on an Illumina GAIIx platform.

Raw sequencing reads were subjected to quality control prior to downstream analysis. Sequencing quality metrics indicated high data reliability, with average Q30 scores of ~91% and GC content of ~51%, consistent with high-quality plant transcriptome data. After adapter trimming and quality filtering, high-quality clean reads were retained for further analysis.

Clean reads were mapped to the tomato reference transcriptome (ITAG 2.3 cDNA dataset derived from the Heinz 1706 genome assembly SL2.40) [47] using CLC Genomics Workbench v9.1. Mapping parameters were set to a minimum length fraction of 0.9, minimum similarity fraction of 0.8, and a maximum of 10 hits per read. Most reads mapped uniquely to the reference transcriptome, indicating high specificity of alignment.

Gene expression levels were quantified as RPKM (reads per kilobase per million mapped reads) to normalize for sequencing depth and gene length. Differential expression analysis between treatments and control (C) was performed using the built-in statistical model in CLC Genomics Workbench, applying Benjamini–Hochberg false discovery rate (FDR) correction, with genes considered differentially expressed at an adjusted *p*-value ≤ 0.05.

To evaluate overall data structure and biological variability, principal component analysis (PCA) and sample-to-sample correlation analysis were performed. PCA demonstrated clear separation between treatment groups, with the first two principal components explaining a substantial proportion of total variance (PC1: 92%, PC2: 5%). Biological replicates showed high reproducibility, as indicated by strong correlation coefficients (R^2^ = 88–99) among replicates. These results confirm the robustness and reliability of the RNA-seq dataset.

Differentially expressed genes were grouped according to their expression patterns across control, first stress, rehydration, and second stress stages using K-means clustering with Manhattan distance as the similarity metric. Multiple clustering resolutions were examined, and 30 clusters were selected empirically to capture diverse transcriptional response patterns while preserving biologically interpretable expression trajectories. Gene Ontology (GO) and pathway enrichment analysis were carried out using “Plant MetGenMAP” (https://bioinfo.bti.cornell.edu/cgi-bin/MetGenMAP/home.cgi, accessed 5 July 2026) [48].

### 4.6. Analysis of Dehydration Stress Memory Genes Using Quantitative Real-Time PCR

At the end of each dehydration treatment, leaf samples were collected, immediately flash-frozen in liquid nitrogen, and stored at −80 °C until RNA extraction. Total RNA was extracted as described previously, and first-strand cDNA synthesis was performed using the GoScript™ Reverse Transcriptase kit (Promega, Madison, WI, USA) according to the manufacturer’s instructions.

Quantitative real-time PCR (qPCR) was performed to validate RNA-seq results using ten selected dehydration stress memory-related genes (Supplementary Table S1). Gene-specific primers were designed for each target gene. The tomato actin gene was used as the internal reference (housekeeping) gene for normalization of gene expression.

qPCR reactions were performed following previously described conditions using a real-time PCR system, and relative gene expression levels were calculated using the 2^−ΔΔCt^ method. Data were presented as mean ± confidence interval (95% CI) [49]. Each reaction included three biological replicates and technical replicates to ensure reproducibility.

## 5. Conclusions

The present study provides evidence that the investigated tomato cultivar develops physiological and transcriptional memory responses under repeated dehydration stress. Recurrent drought exposure induced significant changes in water status, photosynthetic activity, and proline accumulation, accompanied by extensive transcriptome reprogramming. Multiple groups of memory-associated genes were identified, including transcription factors, signaling proteins, osmotic adjustment genes, antioxidant enzymes, and chromatin-related regulators. The results suggest that drought memory in tomato involves the coordinated regulation of signaling pathways, metabolic adaptation, ROS detoxification, and recovery-associated processes. In particular, the enrichment of chromatin remodeling and histone-related genes during the recovery stage indicates that epigenetic mechanisms may play an important role in maintaining transcriptional memory between stress events. The observed expression patterns also demonstrate that tomato ‘Revenant’ balance stress tolerance with energy conservation and growth adjustment during repeated dehydration cycles. Overall, this work provides an initial transcriptomic framework for understanding drought stress memory responses in tomato and identifies candidate genes, which requires validation across multiple genotypes.

Although transcriptomic profiling revealed strong evidence of drought memory, future studies integrating chromatin immunoprecipitation, methylome analysis, and functional validation of candidate genes are required to fully elucidate the epigenetic mechanisms underlying stress memory in tomato. Future studies should employ single-cell or single-nucleus RNA sequencing and spatial transcriptomics to resolve cell-type-specific heterogeneity underlying drought stress memory in tomato [50]. These high-resolution approaches will enable precise mapping of transcriptional memory programs across distinct tissues and better link the identified bulk RNA-seq clusters to specific cellular contexts.

## Figures and Tables

**Table 1:** Recorded expression patterns, memory type, associated genes for all resolve clusters.

Cluster	Expression Pattern (C → S1 → H → S2)	No. of Genes *	Memory Type	Representative Genes/Categories	Functional Enrichment	Proposed Biological Role
**1**	low → moderate → stable → slight decrease	Medium	Basal stress memory	Oxidoreductases, cytochromes	Redox homeostasis, oxidation–reduction	Basal cellular protection and pre-conditioning against oxidative stress
**2**	low → moderate → low → stable	Medium	Early signaling memory	Kinases, RNA polymerase proteins	Signal transduction, transcription regulation	Transient signaling activation during initial drought perception
**3**	low → high → low → high	High	Type I (enhanced response)	WRKY, ROS detox enzymes	ABA signaling, ROS detoxification	Classic transcriptional drought memory with rapid reactivation during S2
**4**	low → moderate → moderate → high	Medium	Metabolic memory	Invertases, sugar metabolism genes	Carbohydrate metabolism, osmotic adjustment	Progressive osmotic adaptation and sugar-based metabolic memory
**5**	low → moderate → higher → highest	Medium	Metabolic memory	Metabolic enzymes	Secondary metabolism, catalytic activity	Cumulative metabolic activation during repeated stress
**6**	low → high → low → moderate	Medium	Type II (attenuated response)	Proteases, kinases	Protein catabolism, stress adaptation	Energy-saving adaptive response during repeated stress
**7**	low → high → low → high	High	Type I (enhanced response)	ERF transcription factors, kinases	Hormonal signaling, transcription regulation	ABA/ethylene-mediated transcriptional memory
**8**	low → moderate → moderate → high	Medium	Metabolic memory	Dehydrogenases	Energy metabolism, organic acid metabolism	Metabolic reprogramming and energy redistribution
**9**	high → low → partial recovery → low	Medium	Growth suppression memory	Photosystem proteins	Photosynthesis, light reactions	Suppression of growth-related pathways during repeated drought
**10**	low → high → low → weaker high	Medium	Type II (attenuated response)	Kinases	Signal transduction	Dampened signaling to reduce metabolic cost
**11**	high → low → high → low	Medium	Recovery-associated memory	Histones, chromatin proteins	Chromatin organization, DNA packaging	Epigenetic memory establishment during recovery
**12**	stable → high → sharp decrease → rebound	Medium	Stress-reset memory	Stress enzymes	Stress response, recovery	Resetting and reactivation of stress-responsive pathways
**13**	low → moderate → higher → highest	Medium	Metabolic memory	Lipid metabolism genes	Lipid metabolism, osmotic balance	Membrane remodeling and osmotic adjustment
**14**	high → low → high → low	Medium	Growth suppression memory	Ribosomal proteins	Translation, ribosome biogenesis	Reallocation of energy from growth toward survival
**15**	low → high → low → weaker high	Medium	Type II (attenuated response)	Proteases	Stress adaptation, proteolysis	Controlled reduction of stress responsiveness
**16**	low → high → low → weak response	Medium	Type II (attenuated response)	Protein degradation enzymes	Cellular response, protein turnover	Adaptive attenuation to minimize energy expenditure
**17**	low → moderate → stable → lower	Medium	Adaptive signaling	Kinase cascade proteins	Signal transduction	Fine-tuning of repeated stress signaling
**18**	low → low → high → moderate	Medium	Recovery-associated memory	DNA repair proteins	DNA repair, chromatin remodeling	Recovery-stage programming and genome stabilization
**19**	low → high → low → higher	High	Type I (enhanced response)	Aquaporins, ROS genes	Water transport, drought response	Physiological drought memory and water regulation
**20**	low → moderate → higher → high	Medium	Metabolic memory	Carbohydrate metabolism genes	Energy balance, carbohydrate metabolism	Osmotic balance and metabolic adaptation
**21**	low → high → partial recovery → altered high	High	Type III (reprogrammed response)	TFs, detoxification enzymes	Stress response, transcription regulation	Primed transcriptional reprogramming
**22**	high → low → highest → low	Medium	Recovery-associated memory	Histones, transport proteins	Chromatin modification, transport	Epigenetic resetting and recovery-associated signaling
**23**	low → moderate → moderate → higher	Medium	Metabolic memory	Metabolic enzymes	Metabolic reprogramming	Energy conservation and biochemical adaptation
**24**	low → low → moderate → high	Medium	Recovery-driven memory	Transporters	Transport, recovery response	Recovery-mediated activation of stress adaptation
**25**	low → high → low → suppressed	Medium	Mixed memory response	ROS detox enzymes	Oxidative stress response	Transitional adaptive response between stress cycles
**26**	stable → moderate → moderate → moderate	Low	General stress response	Stress-related proteins	Cellular stress response	Broad-spectrum stress acclimation
**27**	high → low → slight recovery → low	Medium	Growth suppression memory	Photosynthetic proteins	Photosynthesis, growth	Sustained suppression of energy-consuming processes
**28**	high → low → moderate → low	Medium	Growth suppression memory	Translation-related proteins	Translation, biosynthesis	Long-term repression of growth-related metabolism
**29**	low → moderate → higher → high	Medium	Metabolic memory	Osmotic adjustment genes	Metabolism, osmotic balance	Progressive biochemical adaptation to repeated drought
**30**	low → moderate → high → highest	High	Type I/cumulative memory	TFs, metabolic genes	Transcriptional activation, stress response	Strong cumulative drought memory and sustained activation

* See Figure 3.

**Table 2:** Major dehydration stress memory categories detected in tomatoes along with respected cluster number.

**Memory Category**	**Clusters**
Basal stress memory	1
Early signaling memory	2
Type I enhanced memory	3, 7, 19, 21, 30
Type II attenuated memory	6, 10, 15, 16
Metabolic memory	4, 5, 8, 13, 20, 23, 29
Recovery/epigenetic	11, 18, 22
Growth suppression	9, 14, 27, 28
Stress-reset memory	12
Adaptive signaling	17
Recovery-driven memory	24
Mixed memory response	25
General stress response	26

## Data Availability

The original contributions presented in this study are included in the article/Supplementary Materials. Further inquiries can be directed to the corresponding author.

## Supplementary Materials

The following supporting information can be downloaded at https://www.mdpi.com/article/10.3390/ijms27146187/s1.

## Abbreviations

The following abbreviations are used in this manuscript:

C	Control
S1	First stress
H	Rehydration
S2	Second stress
