# Supplementary material for: Dehydration Stress Memory Genes in Tomato (Solanum lycopersicum L.)
Source: Int J Mol Sci. 2026 Jul 10;27(14):6187. doi: 10.3390/ijms27146187 (PMC13410683; doi:10.3390/ijms27146187)
Supplement: Supplementary file 1 [file ijms-27-06187-s001.zip › ijms-4393966-supplementary.pdf]

International Journal of
Molecular Sciences

MDPI

Supplementary Table S1. List of investigated dehydration stress memory genes in tomatoes and their description and primer sequences used to amplify them using qPCR, the last one corresponds to the reference gene actin.

Durum Locus Number	Gene Description	Forward and Reveres Primers 5′-3′
Solyc00g187050	Leucyl aminopeptidase	AACGTGTTCAAAATTTCAACGGTGG<br>CGTCGCTGCATCATCGCTATTAATC
Solyc02g084850	TAS14	CTGGTGGAGAATATGGAACTCAAGGC<br>CTTCATGTTGTCCAGGCATCTTCTCC
Solyc04g078840	BZIP transcription factor	CACTGAATGTGAATGGGGTCAGATCC<br>CCACTATTTGGGATAGCCATAGGAGC
Solyc06g060760	Aquaporin	AATTACTTGGCTCCACTGTTGCTTGC<br>ACAATAAAACCAATTGCAATGGGTGC
Solyc01g101060	S-adenosylmethionine synthase	TTTGTTCTTTGTTGTTTGCTTGTGGC<br>TCCAAGGCCTAAGATATCCATCCAAC
Solyc03g123500	Ethylene responsive transcription factor 2a	GACCTGTGGTCCTTTGATGATGTTCC<br>ATTCTTCTTCCACCACCAGACACACC
Solyc06g074740	Vacuolar protein sorting-associated protein	TGGAGATACCTCGTCTGAGGGATCG<br>TACTTCATCACTCAAGCAAACACCGC
Solyc01g096190	Calcium-transporting ATPase	AGCCGAGGGTCTTACATTCTGATTCC<br>CTGTGATAGTTCAACAAGCGTGTGGC
Solyc09g005620	Glutaredoxin	TTCCCGAAGGAATCTGGTGTATATGC<br>TGATTTCCAAGATTCAGTTAAGGCGG
Solyc04g009440	NAC domain protein	CAAATTGGATTATGCACGAGTACCGC<br>AAGTAGTCGTTTGCTGGTGTCGATCG
Solyc11g005330	actin	AGGATCCATCCTTGCATCACTTAGC<br>TAATTGCCCTTCTTTCATAGCCCC

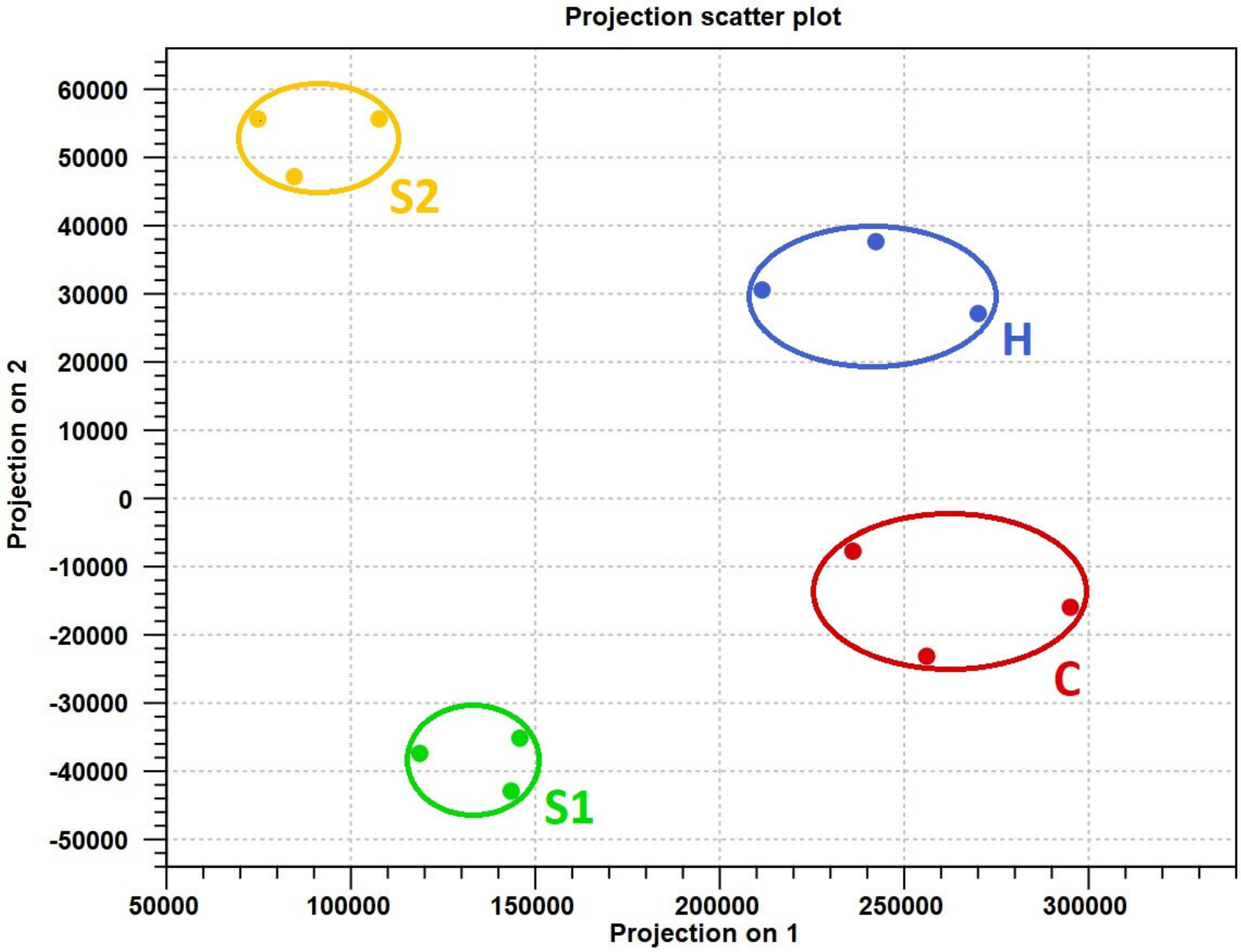

Supplementary Figure S1. PCA for the biological replicates of RNA-seq data in tomato plants in the four consequent stages control (C), first drought stress (S1), rehydration (H) and second drought stress (S2).

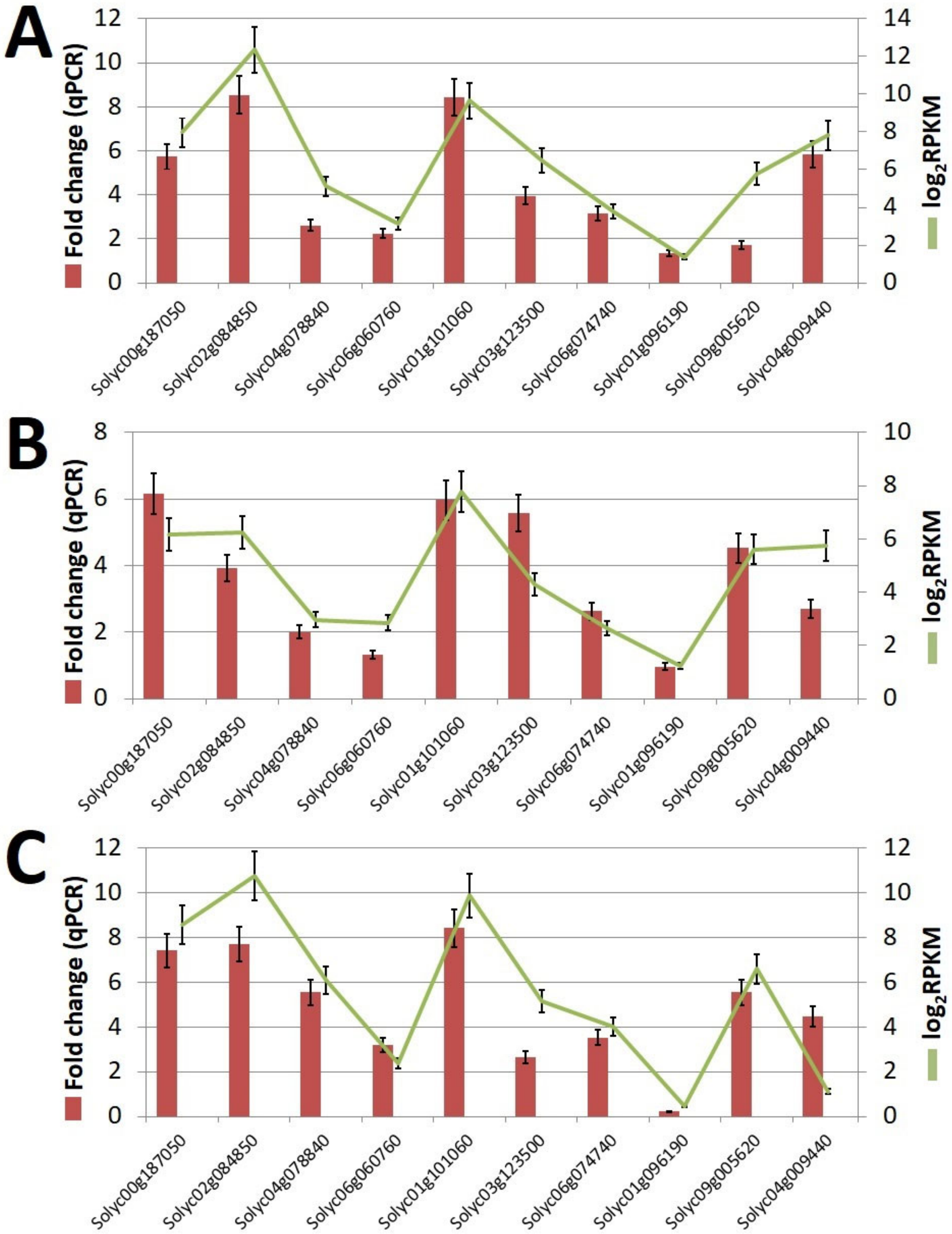

Supplementary Figure S2. RNA-seq validation by quantitative real-time PCR (qRT-PCR). Each graph contains the relative expression level of selected genes in tomatoes. (A) First drought stress, (B) Rehydration, (C) Second drought stress**.** Bars represent data from qRT-PCR, while lines indicate data from RNA-seq.
